# The “two flowers therapy”: clinical efficacy and mechanisms of a total glucosides of paeony -colchicine dual-drugs for Behçet’s disease derived from Chinese physicians’ medication experience

**DOI:** 10.3389/fimmu.2026.1813907

**Published:** 2026-07-21

**Authors:** Can Liu, Lei Li, Qinlong Sun, Yixuan Zhang, Siyao Li, Jian-long Guan, Jianfei Cai

**Affiliations:** 1 Department of Rheumatology and Immunology, Huadong Hospital, Fudan University, Shanghai, China; 2 Clinical Medical College, Fudan University, Shanghai, China; 3 School of Stomatology, Fudan University, Shanghai, China

**Keywords:** Behçet’s disease, colchicine, network pharmacology, total glucosides of paeony, traditional Chinese medicine

## Abstract

**Objective:**

To elucidate the synergistic mechanism of “Two flowers therapy” — a Behçet’s disease (BD) treatment regimen used in China for more than 30 years, namely Total Glucosides of Paeony (TGP)-colchicine — and its efficacy, safety in BD with mucocutaneous involvement.

**Methods:**

A retrospective clinical cohort study integrated with computational biology was performed. Five bioactive components were chosen, among which four were from TGP and one was colchicine; 31 overlapping BD-related targets were identified via multi-omics, finally 6 core genes confirmed. 355 BD patients were divided into combination group (CG, n=231) and monotherapy group (MG, n=124).

**Results:**

Four active components of TGP (oxypaeoniflorin, albiflorin, benzoyl paeoniflorin, paeoniflorin) and colchicine constituted five bioactive compounds. A total of 837 BD-related targets were retrieved from GeneCards, with 31 overlapping targets between the five compounds and BD. A PPI network (31 nodes, 197 edges) was constructed, and core targets (MMP9, ICAM1, FGF2, TLR4, EGFR, NOS3) were identified. Molecular docking confirmed their high affinity: colchicine formed hydrogen bonds with EGFR, ICAM1, NOS3 (2.2–3.4 Å) and hydrophobic interactions with TLR4; TGP components formed 2–4 hydrogen bonds with EGFR, FGF2, MMP9 (2.2–3.9 Å). GO analysis involved inflammation- and immune-related biological processes; KEGG identified 10 enriched pathways (including Lipid and atherosclerosis, AGE-RAGE) regulating inflammation, immunity and vascular function. Clinically, CG had superior early (M1–M2) efficacy: oral ulcer prevalence was 0.0% vs. 21.0% (M1) and 0.0% vs. 100.0% (M2), genital ulcer prevalence 0.9% vs. 6.5% (M1) (all p<0.05). Both groups achieved complete ulcer resolution from M3. ESR in CG was significantly lower at M2 (p<0.001), with no CRP difference. CG had transient diarrhea; no drug-associated cytopenia was reported in either group.

**Conclusion:**

TGP combined with colchicine, a regimen used in China for more than 30 years, exerts therapeutic effects on BD by regulating core targets and inflammatory pathways. Clinically, this regimen more effectively controls early mucocutaneous lesion recurrence in BD patients, with good long-term efficacy and safety.

## 1 Introduction

Behçet’s disease (BD) is a variable vessel vasculitis and chronic inflammatory disease with prominent vascular involvement. Featuring highly heterogeneous multi-organ lesions, it affects mucocutaneous tissues, eyes, gastrointestinal tract, arthrosis, vascular tissues, cardiovascular, nervous, and hematological systems. The disease is classically characterized by oral ulcers, genital ulcers and uveitis. Recurrent painful oral and genital ulcers constitute the most prevalent symptomatic manifestations of BD, which markedly undermine patients’ physical comfort, mental health and overall quality of life (1, 2). For BD management, colchicine remains a first-line agent widely recommended by international guidelines for mucocutaneous manifestations owing to its well-documented anti-inflammatory and anti-neutrophilic activities (3). Colchicine is a tropolone alkaloid originally extracted from *Colchicum autumnale* L. (*C. autumnale*, family Colchicaceae, genus *Colchicum*) nearly two centuries ago (4, 5). It is particularly effective for cutaneous manifestations such as erythema nodosum and genital ulcers and serves as the standard first-line therapy for most BD patients with isolated mucocutaneous involvement (6, 7). In contrast, a large body of clinical practice in China has developed a distinctive treatment strategy that complements mainstream guideline recommendations: the combined administration of total glucosides of paeony (TGP) and colchicine for BD (8). According to abundant ancient Chinese medical literatures, the raw root of *Paeonia lactiflora* Pall. (*P. lactiflora*, family Paeoniaceae, genus *Paeonia*). can be made into Radix Paeoniae Alba and Radix Paeoniae Rubra after different Traditional Chinese Medicine (TCM) processing procedures, which possess different clinical indications. Historically, multiple ancient prescriptions centering on Radix Paeoniae Rubra have been widely applied to treat Behçet’s disease. On the basis of this traditional medicinal experience, total glucosides of paeony (TGP), the major bioactive component of *Paeonia lactiflora* Pall., has been successfully extracted and developed into a modern medicinal preparation by contemporary technological means (9). Notably, this combined regimen, colloquially known as the “Two Flowers Therapy”(Shuang-hua Therapy) among Chinese clinicians—a name derived from the fact that both active pharmaceutical ingredients are extracted from flowering plants: colchicine from *C. autumnale* and TGP from *P. lactiflora*— was first trialed in combination by clinicians in the 1990s and has been consistently and widely utilized in routine clinical settings across China for more than three decades, primarily through inter-physician knowledge sharing and clinical experience accumulation, which has been mostly passed down orally via mentorship among physicians rather than being documented in published papers (8, 10–12).

*P. lactiflora*, a perennial herbaceous plant native to Eastern Asia including China, Korea, and Japan, has been employed in TCM for nearly two thousand years, as first recorded in the Shennong Ben Cao Jing (Divine Farmer’s Materia Medica), the earliest extant comprehensive treatise on TCM compiled during the Eastern Han dynasty (25–220 CE) (13–15). In TCM theory, *P. lactiflora* root has a bitter and slightly sour taste, a neutral nature, and enters the Liver and Spleen meridians. It is traditionally used to nourish blood, regulate menstruation, astringe yin, and alleviate pain, with well-documented effects in promoting blood circulation and dispersing blood stasis (16–18). Phytochemically, PRA contains a rich array of bioactive compounds, including monoterpenoids, monoterpene glycosides, tannins, paeonols, flavonoids, and polysaccharides (19). In particular, monoterpene glycosides, which account for approximately 80-90% of the total bioactive components, are the major characteristic and pharmacologically active compounds of PRA (20–22).

TGP is a standardized modern TCM extract product manufactured from the aqueous-ethanol extract of PRA (15, 23). It is officially approved by the National Medical Products Administration of China (Approval Number H20055058) and mainly composed of four major bioactive monoterpene glycosides: oxypaeoniflorin, albiflorin, benzoyl paeoniflorin and paeoniflorin (24). Rooted in TCM theory, TGP possesses the classical effects of clearing heat, cooling blood, and resolving blood stasis, which precisely targets the core heat-toxin and stasis pathogenesis underlying recurrent inflammatory and mucosal lesions in BD (16, 17). Accumulated clinical evidence has proven its value in treating various inflammatory disorders, such as rheumatoid arthritis (RA), systemic lupus erythematosus (SLE), and liver diseases (25, 26). Extensive preclinical and clinical studies have demonstrated that TGP exerts multifaceted pharmacological effects, including potent anti-inflammatory (27, 28), antioxidative (29) and immunoregulatory activities (30). Specifically, TGP has been shown to inhibit pro-inflammatory cytokine production, regulate T-cell subsets, suppress neutrophil activation, and modulate NF-κB and MAPK signaling pathways (31–34). Owing to these properties, TGP has achieved remarkable therapeutic efficacy and excellent safety/tolerance profile in these inflammatory diseases. However, despite the fact that TGP is routinely prescribed in combination with colchicine for BD by the vast majority of rheumatologists in China, only a handful of small-scale clinical studies have preliminarily explored the efficacy of TGP monotherapy or combination therapy in BD (35), and the precise molecular mechanisms underlying its synergistic effects with colchicine remain almost completely unexplored.

Currently, there is no universally accepted standardized therapeutic regimen for BD, as clinical management remains highly individualized and dependent on the specific organ involvement and disease severity (36, 37). While colchicine monotherapy is effective for many patients with mild mucocutaneous BD, a significant proportion of patients experience incomplete response, treatment resistance, or adverse effects such as gastrointestinal intolerance (38–41). This unmet clinical need has driven the search for more effective and better-tolerated combination therapies. Considering that both colchicine and TGP have demonstrated efficacy in reducing BD-related inflammation and relieving mucocutaneous symptoms, and that their distinct mechanisms of action may confer synergistic therapeutic effects, we therefore hypothesized that the dual-drug combination regimen of colchicine and TGP—officially designated as the “Two Flowers Therapy” in this study based on its widespread clinical nomenclature—could serve as a superior and safer therapeutic strategy for BD patients with mucocutaneous involvement. This hypothesis is supported by over three decades of real-world clinical experience in China, where this combination has been empirically shown to provide better symptom control, fewer relapses, and reduced adverse effects compared to colchicine monotherapy (10, 11, 35).

Hence, we designed this dual-therapy regimen comprising colchicine and TGP, and our study aimed to systematically evaluate the efficacy and safety of this regimen in BD patients with mucocutaneous involvement. Additionally, we further explored the potential underlying mechanisms of this “Two Flowers therapy” through network-based multi-omics technologies (42).

## 2 Materials and methods

### 2.1 Mechanism research

#### 2.1.1 Screening for active components and target proteins

**Figure 1:**
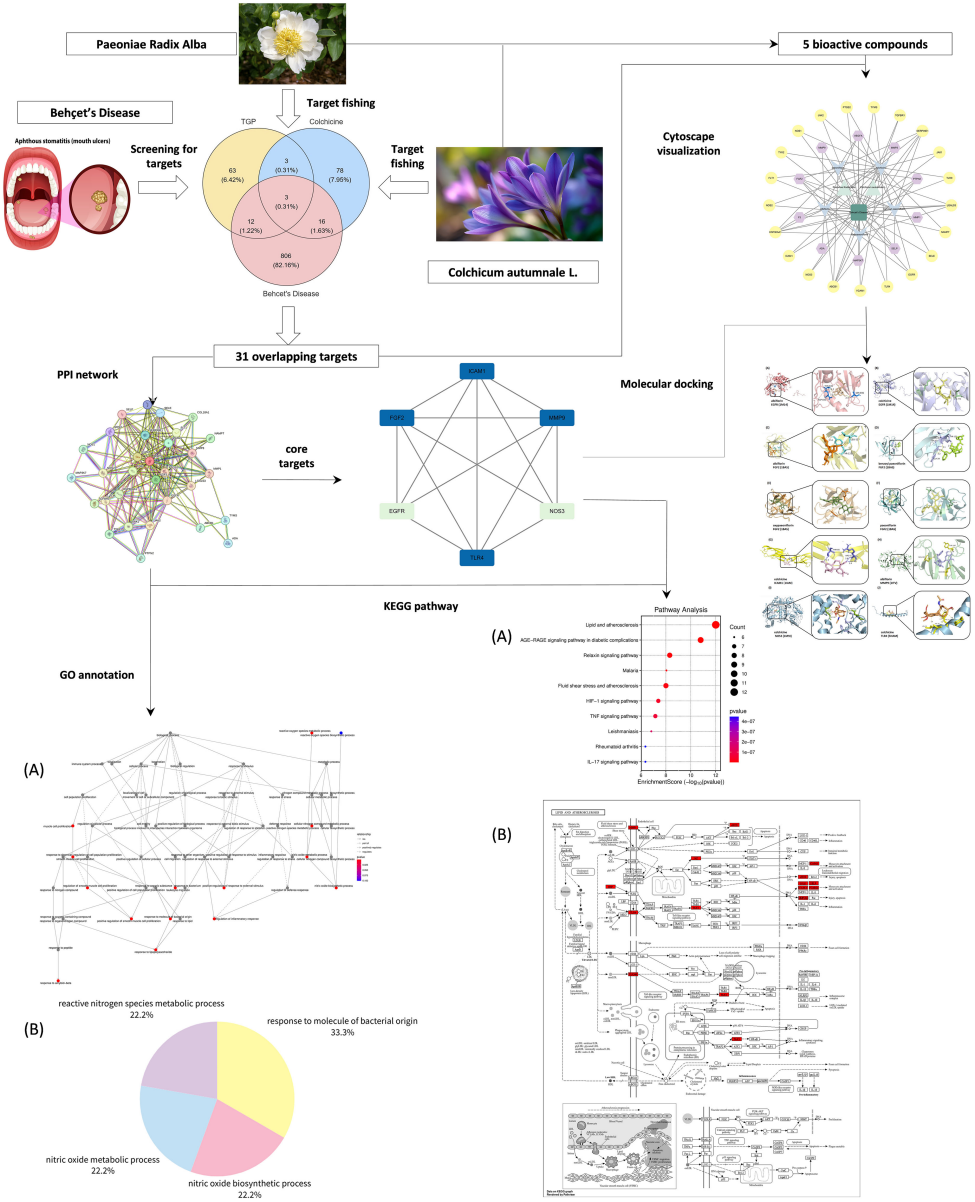
Workflow of the study design.

Four major bioactive constituents of TGP, namely oxypaeoniflorin, albiflorin, benzoyl paeoniflorin and paeoniflorin, were screened and validated via the Traditional Chinese Medicine Systems Pharmacology Database and Analysis Platform (TCMSP, https://tcmsp-e.com/tcmsp.php). We then obtained the Simplified Molecular Input Line Entry System (SMILES), a universal notation for describing molecular structures, of each compound from Pubchem Database (https://pubchem.ncbi.nlm.nih.gov). Related target genes were further predicted using Swiss Target Prediction (https://swisstargetprediction.ch). The overall workflow of the study design is shown in **Figure 1**.

#### 2.1.2 Collection of Behçet’s disease related targets

BD related targets were searched and collected on the platform of Genecards Database (https://www.genecards.org/), a database that provides comprehensive information on all annotated and predicted human genes (43). Then we mapped the BD related targets and the targets of TGP as well as colchicine in the Gene Denovo platform (https://www.genedenovo.com), resulting in a Venn diagram with 31 overlapping targets.

#### 2.1.3 Identifying core targets

The Gene Expression Omnibus (GEO, http://www.ncbi.nlm.nih.gov/geo/) is an international public repository for high-throughput microarray and next-generation sequence functional genomic data sets submitted by the research community (44). Here we analyzed differentially expressed genes between healthy people and BD patients with GEO2R in a correlated gene research (Series Accession: GSE209567) of 44 samples with 29 BD patients and 15 health controls, resulting in 6 core genes. The P-value was less than 0.05 for better accuracy (45).

#### 2.1.4 PPI network and visualization

Subsequently, we put the 31 overlapping targets into the STRING network platform (https://string-db.org/) so as to figure out the connection among them. Constructing a protein-protein interaction (PPI) network, we set the Organisms to Homo sapiens with the confidence value 0.4 (medium) (46, 47). Therefore, 31 overlapping targets and 5 related bioactive compounds were visualized in Cytoscape 3.10.4. In the meantime, we ranked the 6 core targets by Degree method in Cytoscape 3.10.4, as well as displayed them in circle with the shade of color depending on the magnitude of their values (48).

#### 2.1.5 Molecular docking

To prepare the small-molecule ligands and protein receptors, we acquired the *Mol2 format structures of bioactive compounds from the TCMSP Database and retrieved the initial *PDB format 3D protein structures from the PDB Database. Protein structures were further processed using PyMol software to eliminate water molecules and native ligands at active sites. The purified proteins were then hydrogenated and converted to *pdbqt format via AutoDock software. Meanwhile, we defined the rotatable bonds of small molecules and exported the files in *pdbqt format. Ultimately, molecular docking of the processed ligands and receptors was performed using AutoDock Vina. The grid box was set to cover the whole favorable protein binding site with different size and X, Y, Z centers in different target protein. All spacing value of the grid box was 1.000Å. For EGFR(1M14), the grid box size of 96.0×65.0×51.0 points was centered at (6.259, 7.298, 58.125) Å, while for FGF2 (1BAS) the grid box size of 38.0×35.0×35.0 points was centered at (2.157, 5.451, -1.961) Å. The grid box size of 71.0×51.0×54.0 points for ICAM1 (1IAM) was centered at (38.913, 78.038, 3.418) Å and the grid box size of 75.0×76.0×47.0 points for MMP9 (1ITV) was centered at (-26.26, -33.776, -13.002) Å. Size of 74.0×75.0×87.0 points, the grid box was centered at (19.03, 10.677, 36.253) Å for NOS3 (1M9J). Likewise, for TLR4 (5NAM), the grid box size of 35.0×40.0×61.0 points was centered at (4.202, 7.461, -26.956) Å. Eventually, every consequence of specific ligand to receptor was visualized in PyMol software.

#### 2.1.6 GO annotation

**Figure 2:**
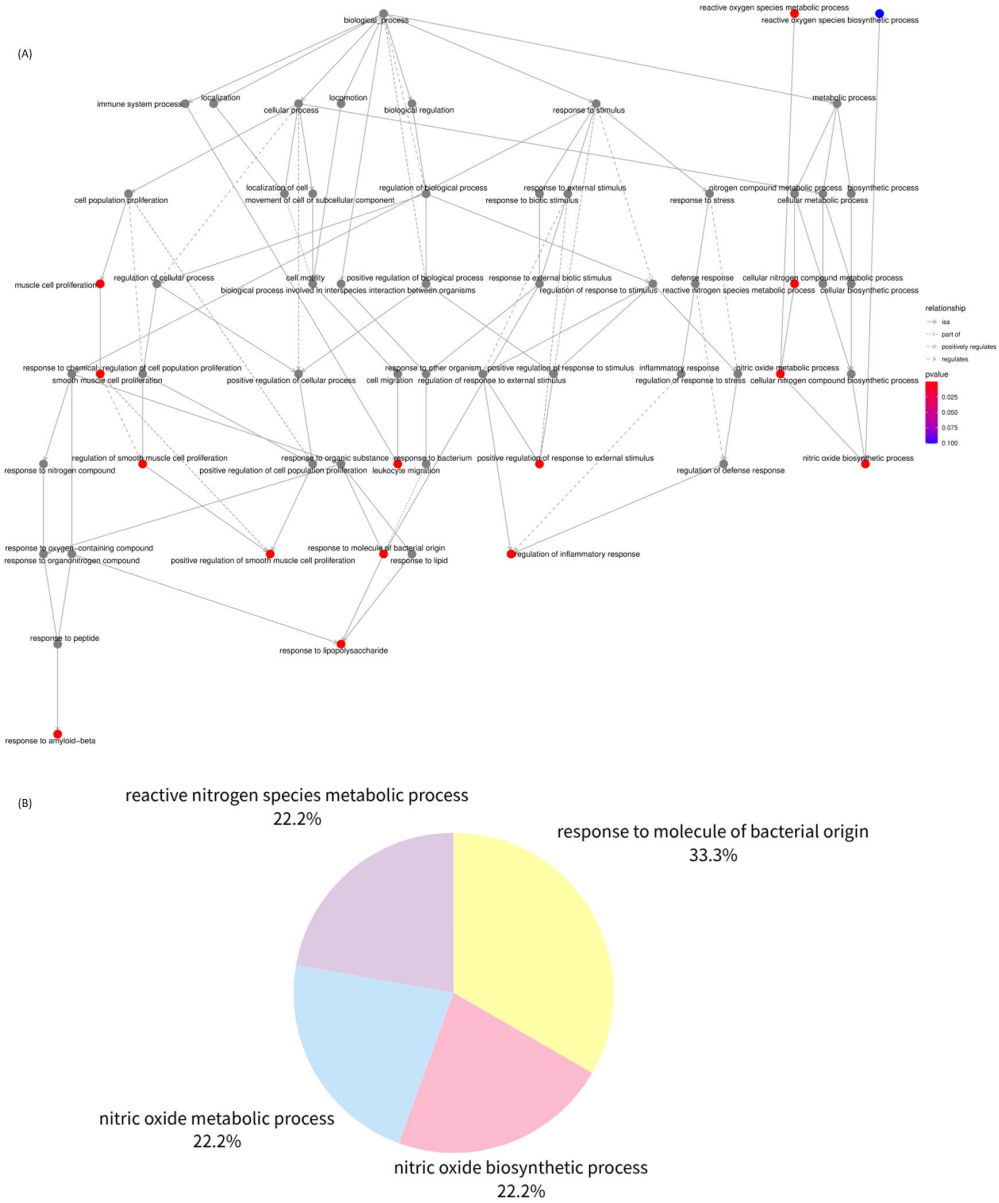
**(A, B)** GO enrichment analysis of key targets of TGP and colchicine in treatment of BD. **(A)** Network visualization of the significantly enriched biological processes. **(B)** Pie chart displaying the proportion of each key biological process.

This study used the Microbioinformatics online platform (https://www.bioinformatics.com.cn/) to perform Gene Ontology (GO) enrichment analysis. It helped us understand the main biological processes of the target genes (49, 50). We uploaded a list of 31 gene symbols and their P-values to the platform. The analysis was set for humans, and we only kept results with a P-value less than 0.05 (51, 52). The platform completed the enrichment analysis in approximately 180 seconds. For the pie chart (**Figure 2B**), to ensure clarity and avoid overcrowding, only the top 4 most enriched biological processes were selected for visualization.

#### 2.1.7 KEGG pathway

**Figure 3:**
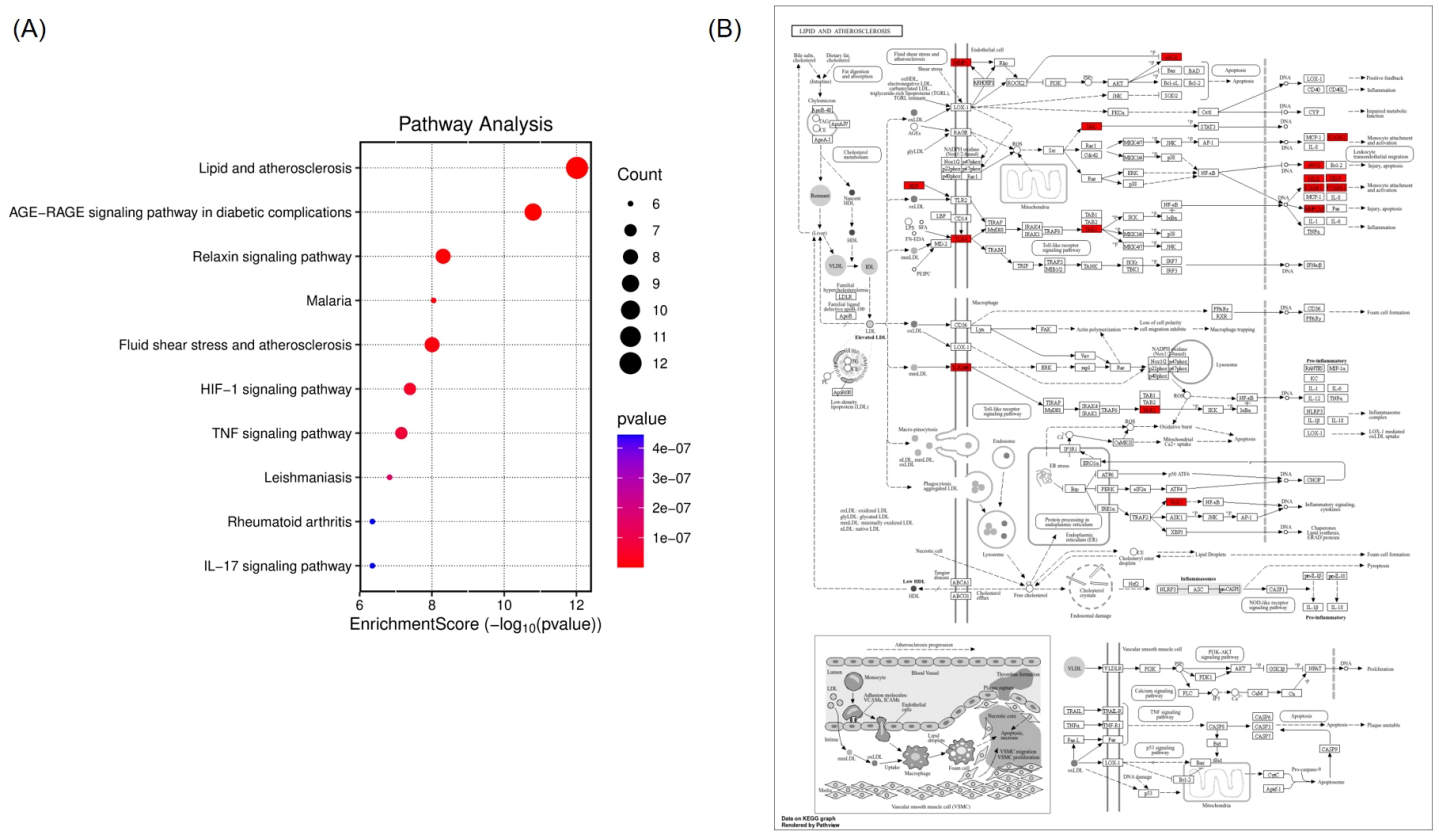
KEGG analysis of key targets of TGP and colchicine in treatment of BD. **(A)** Bubble plot of top 10 KEGG pathways. **(B)** Reprinted of Lipid and atherosclerosis signaling pathway download from the Microbioinformatics platform.

This study also used the Microbioinformatics platform (49, 50) (https://www.bioinformatics.com.cn/) for KEGG pathway analysis. We uploaded the same gene list as in the GO analysis. The settings were the same: human species and P-value less than 0.05 (52). The platform completed the analysis in approximately 180 seconds. The bubble chart (**Figure 3A**) was directly generated by the platform to visualize the top 10 significantly enriched pathways. For the key pathway diagram (**Figure 3B**), the most significantly enriched pathway, namely the “ Lipid and atherosclerosis”, was extracted from the platform’s results (53).

### 2.2 Clinical validation

#### 2.2.1 Patients

A total of 355 newly diagnosed BD patients with mucocutaneous involvement were recruited in this study. All patients were diagnosed according to the International Criteria for BD (54). The included subjects were required to be older than 18 years and present with oral ulceration and/or genital ulceration (55). Patients were excluded if they had thrombosis, arterial disease involvement, gastrointestinal disease involvement, nervous system disease involvement, or joint disease involvement at diagnosis. Additionally, individuals with severe infections, malignancies, or those who were pregnant or lactating were also excluded. **Table 1** shows the basic information of the patients. The ethics board of our hospital approved this study, and all patients signed consent forms.

**Table 1:** Baseline characteristics of BD patients.

Items	BD patients (N = 355)
Age (years), M ± SD	37.5 ± 12.8
Gender, no. (%)	
Female	218 (61.4)
Male	137 (38.6)
Duration of oral ulceration (years), M ± SD	7.1 ± 7.4
History of uveitis, no. (%)	29 (8.2)
Clinical manifestation, no. (%)	
Mucocutaneous involvement	
Oral ulceration	355 (100.0)
Genital ulceration	355 (100.0)
Erythema nodosum	71 (20.0)
Eye involvement	
Uveitis	0 (0.0)
Thrombosis	0 (0.0)
Arterial disease involvement	0 (0.0)
Nervous system disease involvement	0 (0.0)
Biochemical indexes, M ± SD	
WBC (×10^9^/L)	5.7 ± 1.5
Neutrophil (%)	56.6 ± 8.0
RBC (×10¹²/L)	3.9 ± 0.5
Hb (g/L)	139.5 ± 13.6
PLT (×10^9^/L)	210.0 ± 58.5
CRP (mg/L)	10.1 ± 9.2
ESR (mm/h)	24.9 ± 18.8
Scr (μmol/L)	61.6 ± 13.2
BUN (mmol/L)	5.2 ± 1.2
ALT (U/L)	21.0 ± 13.2
AST (U/L)	22.9 ± 7.9
Treatment at baseline, no. (%)	
Colchicine	355 (100.0)
Total Glucosides of Paeony	0 (0.0)

ALT, alanine aminotransferase; AST, aspartate aminotransferase; BD, Behcet’s disease; BUN, blood urea nitrogen; CRP, C‐reactive protein; ESR, erythrocyte sedimentation rate; Hb, hemoglobin; M ± SD, mean ± standard deviation; PLT, blood platelet; RBC, red blood cell; Scr, serum creatinine; WBC, white blood cells.

#### 2.2.2 Treatment process and regimens

**Figure 4:**
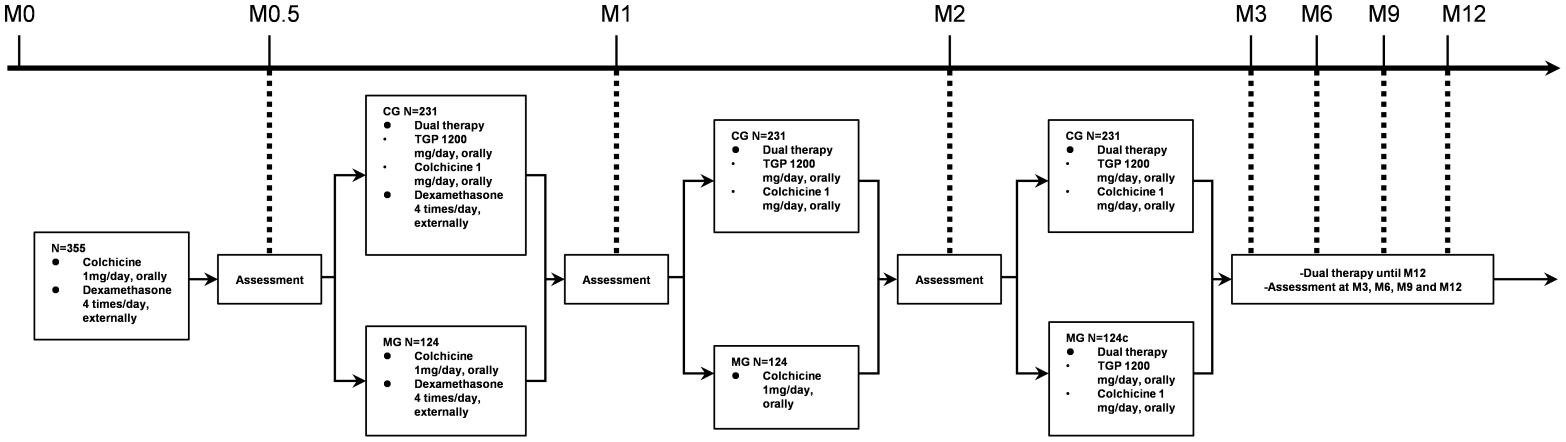
Treatment process.

After joining the study, all patients first received Local topical application of dexamethasone and colchicine for two weeks. The mucocutaneous lesions of all participants after this two-week pretreatment are summarized in **Table 2**. Then, at the two-week point (M0.5), we split them into two groups based on their treatment. Group allocation was determined by patient willingness and physician’s clinical judgment, with no strict randomization. Eligible patients who preferred combined therapy were assigned to the CG, while those who preferred colchicine monotherapy entered the MG. The combination group (CG, n=231) took colchicine (1 mg/day) plus TGP (1200 mg/day) from M0.5 until month 12 in accordance with the TCM “Two Flowers Therapy” regimen for BD as mentioned earlier. The monotherapy group (MG, n=124) continued with only colchicine (1 mg/day) from M0.5 to month 2. After that, from month 3 to month 12, they also switched to the colchicine and TGP combination. During the treatment, doctors could adjust the drug dose if needed, depending on the patient’s condition and any side effects (54). The treatment process is illustrated in **Figure 4**.

**Table 2:** Clinical manifestations after two‐week treatment .

Items	Patients (N = 355)
Oral ulceration, no. (%)	231 (65.1)
Genital ulceration, no. (%)	227 (63.9)
Erythema nodosum, no. (%)	14 (3.9)
Thrombosis, no. (%)	0 (0.0)
Arterial disease involvement	0 (0.0)
Nervous system disease involvement, no. (%)	0 (0.0)

#### 2.2.3 Data collection and assessments

We recorded patients’ basic information and medical history at the start. We checked their clinical symptoms at different time points: M0, M0.5, M1, M2, M3, M6, M9, and M12. These symptoms included oral ulcers, genital ulcers, erythema nodosum, uveitis, thrombosis, arterial disease involvement, and nervous system disease involvement. We also took blood samples to measure lab values at M0, M1, M2, M3, M6, M9, and M12. These included blood cell counts, C-reactive protein (CRP), erythrocyte sedimentation rate (ESR), and tests for liver and kidney function. We also carefully recorded any side effects from the drugs during the study.

#### 2.2.4 Statistical analysis

Continuous data were described as mean and standard deviation. Categorical data were described as count and percentage. Comparisons between the two groups were determined by Student’s t-test, Chi-square test, or Fisher’s exact test, as appropriate. All statistical analyses were performed using IBM SPSS Statistics 26.0, and figures were plotted using GraphPad Prism 10.1.2. A p value of less than 0.05 was considered statistically significant.

## 3 Results

### 3.1 Mechanism result

#### 3.1.1 Screening for active components and target proteins of TGP and colchicine in treatment of BD

**Figure 5:**
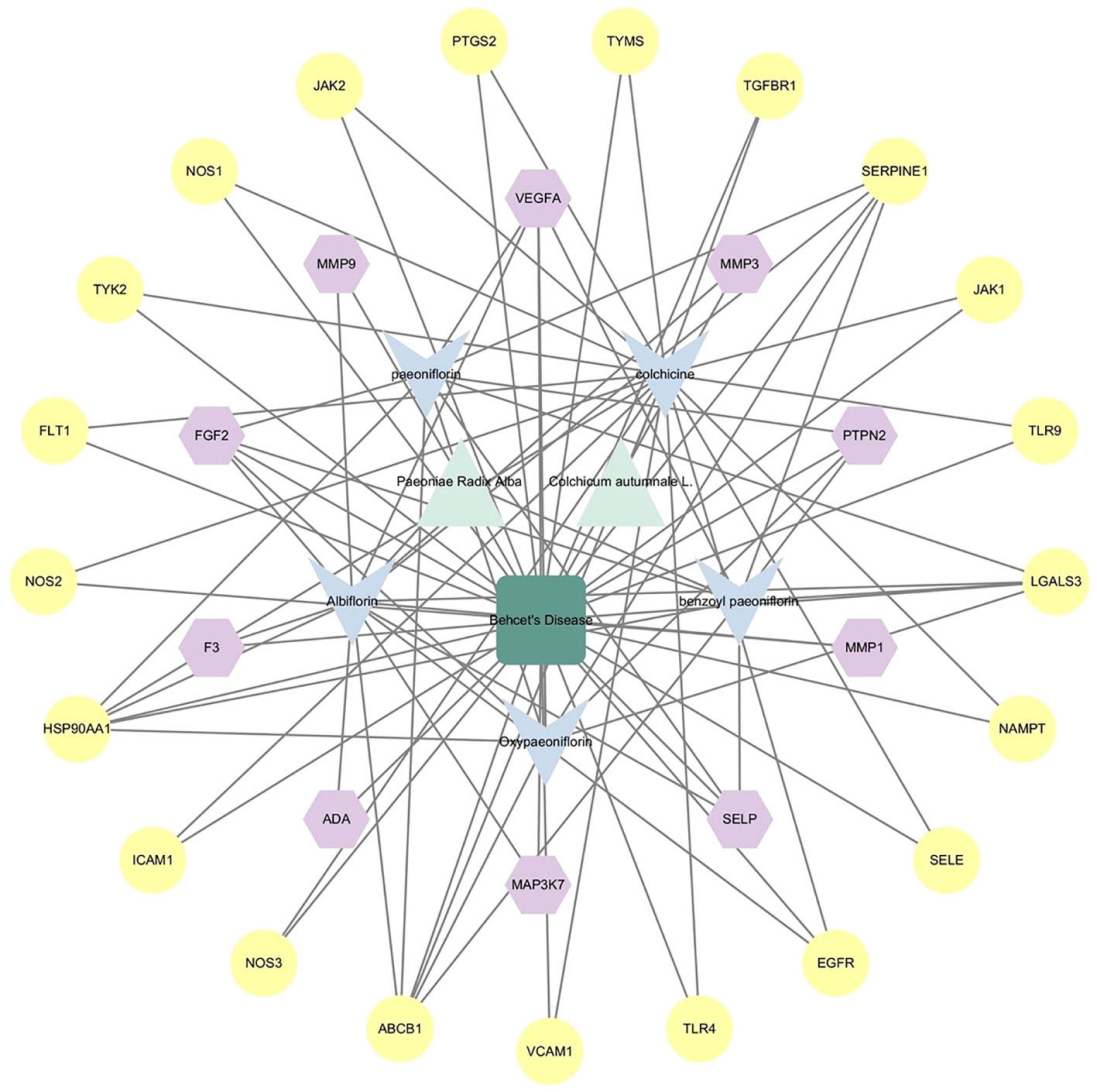
Bioactive compounds and corresponding targets network of TGP and colchicine in treatment of BD.

Oxypaeoniflorin, albiflorin, benzoyl paeoniflorin, paeoniflorin, the bioactive compounds of TGP were screened from TCMSP as well as comprehensive literature (56). Meanwhile, 837 targets of BD were collected from Genecards Database. Visualized in a Venn diagram, the 31 overlapping targets between TGP as well as colchicine and BD were put into Cytoscape along with 5 valid compounds. Hence, we obtained a Concentric Circle Diagram (**Figure 5**).

#### 3.1.2 Construction of PPI network and core targets

**Figure 6:**
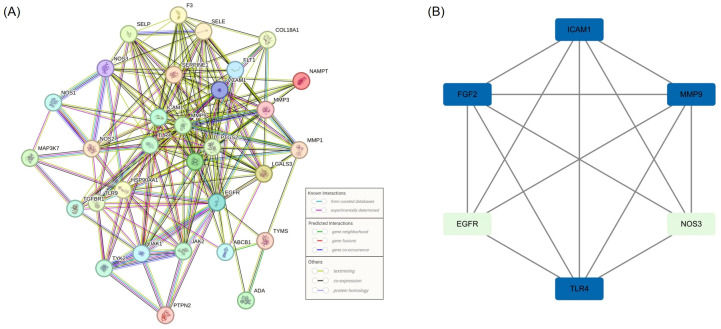
Construction of PPI network and core targets. **(A)** PPI network of potential targets of TGP and colchicine in treatment of BD. **(B)** Core targets of overlapping targets.

Then we input the 31 overlapping targets and the 5 bioactive compounds into the STRING Database. The PPI network has 31 nodes and 197 edges, with average node degree of 12.7 (**Figure 6A**). Selected from GEO Database, the final 6 core genes were imported into Cytoscape for an outcome of a Concentric Circle Diagram with the shade of color depending on the magnitude of their values. In conclusion, the highest value of degree was five, corresponded with MMP9, ICAM1, FGF2 and TLR4, nevertheless, the lowest score was four, related with EGFR and NOS3. (**Figure 6B**).

#### 3.1.3 Validation of candidate TGP and colchicine targets in treating BD

**Figure 7:**
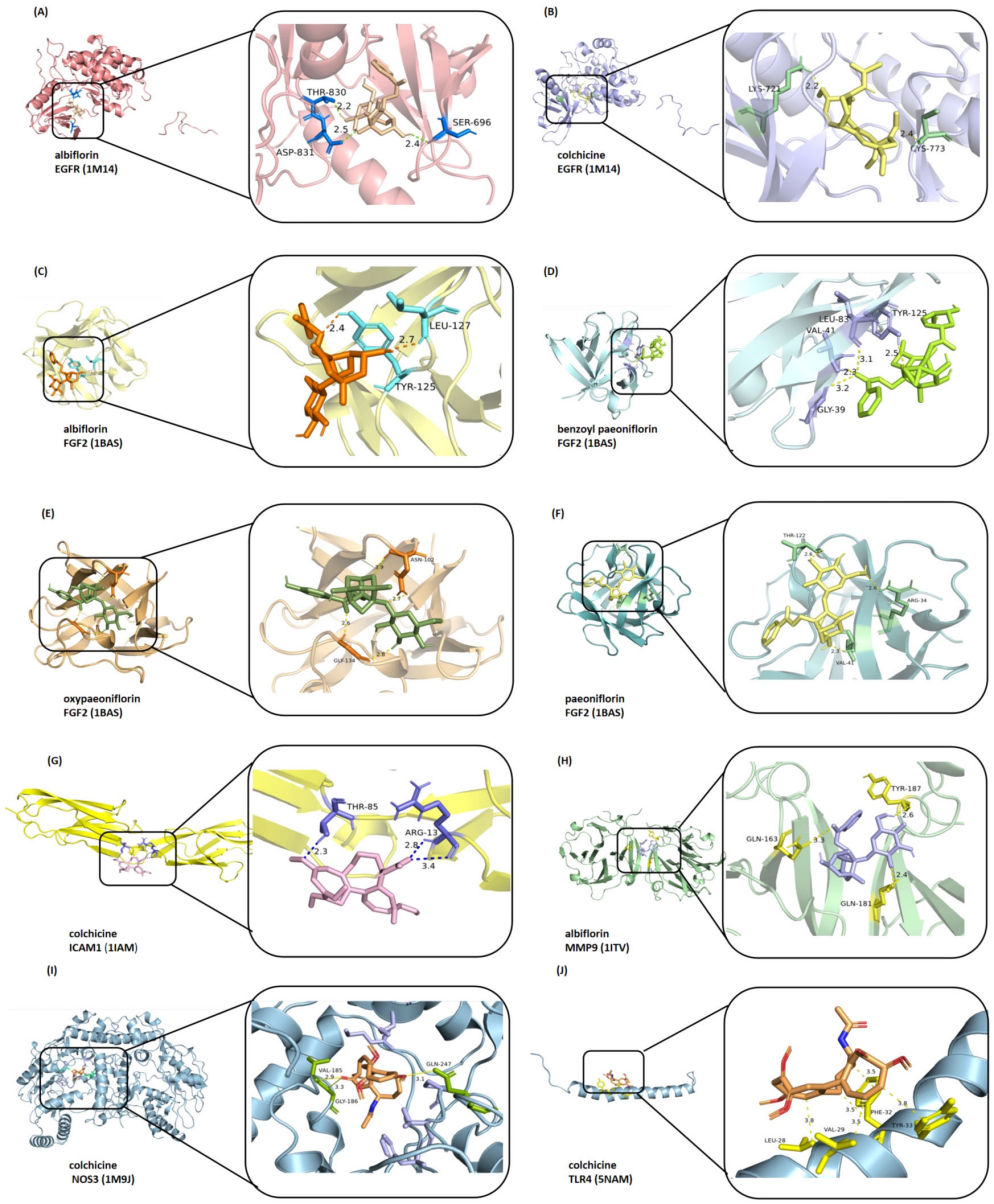
Molecular docking diagrams of BD related targets with main compounds of TGP and colchicine. Albiflorin binds to protein **(A)** EGFR (1M14), **(C)** FGF2 (1BAS) and **(H)** MMP9 (1ITV). Benzoyl paeoniflorin is shown interacting with **(D)** FGF2 (1BAS). Oxypaeoniflorin shows high affinity with FGF2 (1BAS) **(E)**. Paeoniflorin binds to protein **(F)** FGF2 (1BAS). Colchicine is attracted to **(B)** EGFR (1M14), **(G)** ICAM1 (1IAM), **(I)** NOS3 (1M9J) and **(J)** TLR4 (5NAM).

To confirm validation of these bioactive compounds of TGP and colchicine in treating BD, we explored the conformational stability of each couple of ligand and receptor. Confronted with the visualization of molecule docking, we learned that colchicine bound to EGFR (1M14) by 2 hydrogen bonds between it and LYS-721, CYS-773, with bonds length of 2.2 and 2.4 Å, respectively, while it bound to ICAM1 (1IAM) by 3 hydrogen bonds, one between it and THR-85 lengthened 2.3 Å, another two between it and ARG-13 lengthened 2.8 and 3.4 Å respectively. In the meantime, it also formed 3 hydrogen bonds with NOS3 (1M9J) between it and VAL-185, GLY-186, GLN-247 with the bonds length of 2.9, 3.3, 3.1 Å. However, colchicine was attracted to TLR4 (5NAM) not by hydrogen bonds but by hydrophobic interaction between it and LEU-28 (lengthened 3.8 Å), VAL-29 (lengthened 3.5 Å), PHE-32 (both hydrophobic interaction lengthened 3.5 Å)) and TYR-33 (lengthened 3.8 Å). Albiflorin formed 3 hydrogen bonds with EGFR (1M14) between it and THR-830, SER-696, ASP-831 with the bonds length of 2.2, 2.4, 2.5 Å. When the target was FGF2 (1BAS), it could form 2 hydrogen bonds between it and TYR-125, LEU-127, length of 2.4, 2.7 Å. Moreover, it could also bound to MMP9 (1ITV) by 3 hydrogen bonds, length of 2.4, 2.6, 3.3 Å, between it and GLN-181, TYR-187, GLN-163. Similarly, benzoyl paeoniflorin was gravitated to FGF2 (1BAS) by 4 hydrogen bonds, between it and TYR-125, LEU-83, VAL-41, GLY-39, length of 2.5, 3.1, 2.3, 3.2 Å. 4 hydrogen bonds were also formed by Oxypaeoniflorin with FGF2 (1BAS), with 2 hydrogen bonds between it and ASN-102, length of 3.9 and 2.7 Å, 2 hydrogen bonds remaining between in and GLY-134, length of 2.6 and 2.8 Å. Likewise, paeoniflorin bound to FGF2 (1BAS) by 3 hydrogen bonds between it and THR-122, ARG-34, VAL-41, with bonds length of 2.6, 2.6, 2.3 Å. All the illustrations were assembled in **Figure 7**.

#### 3.1.4 GO enrichment analysis results

Based on the 31 target genes obtained from the prior PPI network analysis, GO biological process enrichment analysis was performed using the Microbioinformatics online platform. A total of 1283 biological processes were screened under the threshold of P < 0.05. The visual results of the GO biological process enrichment analysis are shown in **Figure 2**: **Figure 2A** shows the relationships between target genes and enriched biological processes, which were directly output by the analysis platform. **Figure 2B** presents the proportion of each core biological process. The top enriched functional terms mainly involve responses to bacterial-derived molecules, nitric oxide turnover and reactive nitrogen species metabolism. These pathways are functionally correlated with inflammatory reactions and immune modulation.

#### 3.1.5 KEGG pathway enrichment results

KEGG analysis of the 31 genes showed 10 significantly enriched pathways. The top 10 enriched pathways were visualized in a bubble plot **Figure 3A** using the platform’s built-in function. The most enriched pathways were Lipid and atherosclerosis, AGE-RAGE signaling pathway in diabetic complications, Relaxin signaling pathway, Fluid shear stress and atherosclerosis, HIF-1 signaling pathway, TNF signaling pathway, Malaria, Leishmaniasis, Rheumatoid arthritis, and IL-17 signaling pathway. These pathways are linked to inflammation, immunity, and vascular function. The most significantly enriched pathway, the “Lipid and atherosclerosis”, was selected for visualization (**Figure 3B**).

### 3.2 Clinical result

#### 3.2.1 Evaluation of clinical efficacy

Over the 12-month observational period, a rigorous comparison of therapeutic outcomes for mucocutaneous lesions was conducted between the two study groups, with key findings summarized in **Table 3**. Notably, at study enrollment, all patients received a standardized two-week initial treatment regimen comprising topical dexamethasone combined with colchicine to ensure complete ulcer healing before formal group allocation and subsequent follow-up.

**Table 3:** Comparison of clinical manifestations between MG and CG.

Group	MG (N = 124)			CG (N = 231)		
Items	M1 no. (%)	M2 no. (%)	M3 no. (%)	M1 no. (%)	M2 no. (%)	M3 no. (%)
Oral ulceration	26 (21.0)	124 (100.0)	0 (0.0)	0 (0.0)*	0 (0.0)*	0 (0.0)
Genital ulceration	8 (6.5)	0 (0.0)	0 (0.0)	2 (0.9)*	0 (0.0)	0 (0.0)
Erythema nodosum	0 (0.0)	0 (0.0)	0 (0.0)	2 (0.9)	0 (0.0)	0 (0.0)
Uveitis	0 (0.0)	0 (0.0)	0 (0.0)	0 (0.0)	0 (0.0)	0 (0.0)
Thrombosis	0 (0.0)	0 (0.0)	0 (0.0)	0 (0.0)	0 (0.0)	0 (0.0)
Arterial disease involvement	0 (0.0)	0 (0.0)	0 (0.0)	0 (0.0)	0 (0.0)	0 (0.0)
Nervous system disease involvement	0 (0.0)	0 (0.0)	0 (0.0)	0 (0.0)	0 (0.0)	0 (0.0)

Comparison was determined by Chi‐square test or Fisher’s exact test. Boldface and *represented p value < 0.05. MG, monotherapy group, colchicine to dual‐therapy; CG, combination group, sustained dual‐therapy.

Since colchicine monotherapy exhibited unsatisfactory efficacy for oral ulcers in Chinese patients, all patients were switched to colchicine combined with TGP therapy starting from the third month.

Subsequent efficacy assessments focused on recurrence rates of oral and genital ulcers, as well as the resolution of erythema nodosum. Analysis of oral ulcer recurrence revealed prominent intergroup differences during the early treatment phase: at the M1 and M2 follow-up time points, the CG exhibited significantly lower oral ulcer recurrence rates compared with the MG (M1: 0.0% vs. 21.0%, p < 0.05; M2: 0.0% vs. 100.0%, p < 0.05). A similar favorable trend was observed for genital ulcer recurrence at M1, with a markedly lower recurrence rate in the CG relative to the MG (0.9% vs. 6.5%, p < 0.05).

From the 3-month follow-up onward, both groups achieved and maintained complete remission of oral and genital ulcers, with no statistically significant differences between the two groups. Throughout the entire study period, the resolution of erythema nodosum was comparable between the CG and MG, with no obvious intergroup variations.

#### 3.2.2 Analysis of laboratory biomarkers

**Figure 8:**
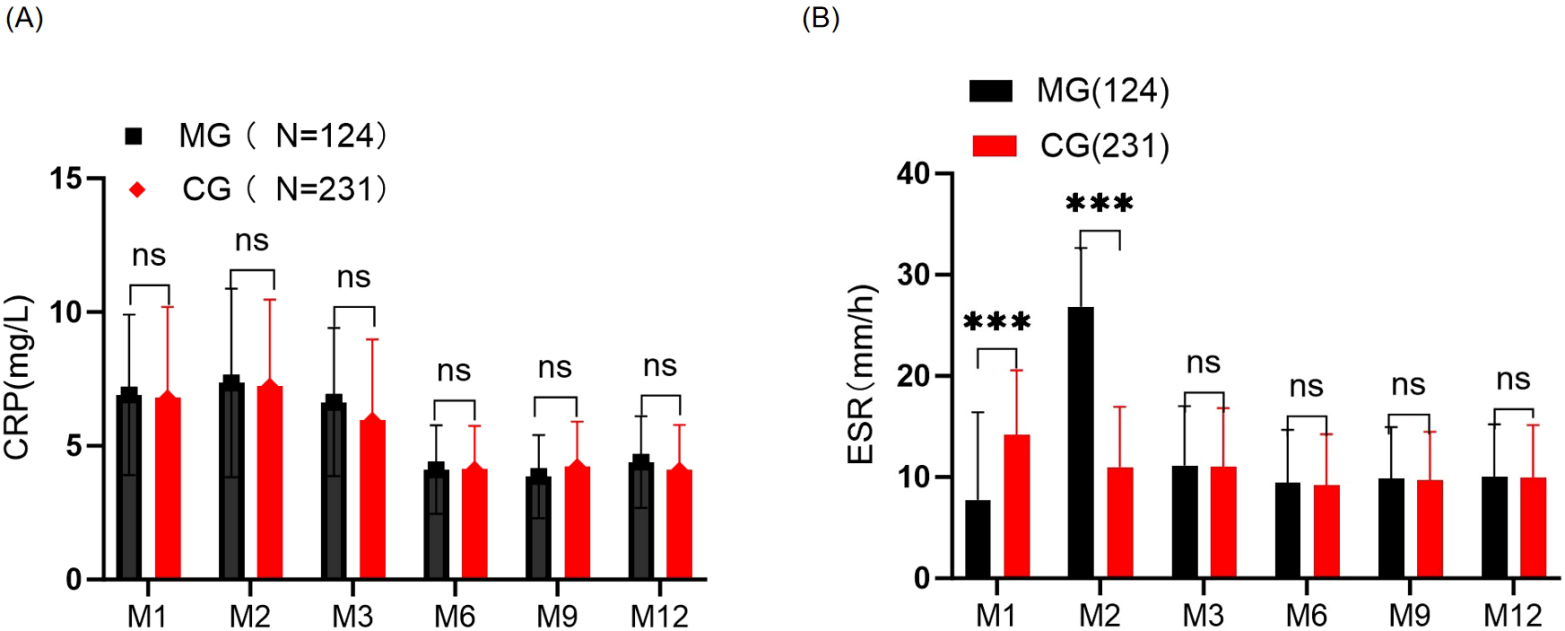
CRP and ESR in MG and CG. Comparison of CRP **(A)** and ESR **(B)** at different time points between MG and CG. CRP, C‐reactive protein; ESR, erythrocyte sedimentation rate; MG, monotherapy group; CG, combination group.

The trajectory of the inflammatory marker ESR differed notably between the regimens. While the CG displayed an initially higher ESR value at M1, a precipitous decline was observed, resulting in a value significantly inferior to that of the MG by the M2 visit (p < 0.001). After M3, ESR levels equilibrated and remained comparable between the cohorts for the study’s duration (**Figure 8B**). In contrast, serum CRP concentrations showed no significant inter-group differences at any measured interval. Other hematological and biochemical parameters, including complete blood count and indicators of hepatic and renal function, consistently remained within normative limits for participants in both arms, notwithstanding sporadic fluctuations that reached statistical significance (**Figure 8A**). Detailed longitudinal data of all biochemical parameters are provided in **Table 4**.

**Table 4:** Comparison of biochemical indexes between the CG and MG.

Group	MG (N = 124)			CG (N = 231)		
Items	M1 M ± SD	M2 M ± SD	M3 M ± SD	M1 M ± SD	M2 M ± SD	M3 M ± SD
WBC (×10^9^/L)	5.5 ± 1.4	6.1 ± 1.5	6.5 ± 1.5	5.6 ± 1.4	6.5 ± 1.6*	6.3 ± 1.5
Neutrophil (×10^9^/L)	55.0 ± 8.0	61.7 ± 7.5	65.3 ± 5.5	55.3 ± 7.9	62.7 ± 6.9	65.2 ± 5.8
RBC (×10¹²/L)	4.4 ± 0.5	4.1 ± 0.3	4.1 ± 0.3	4.4 ± 0.4	4.0 ± 0.3*	4.1 ± 0.3
Hb(g/L)	138.6 ± 16.6	133.3 ± 9.7	132.5 ± 13.0	139.1 ± 14.0	133.4 ± 10.1	132.6 ± 12.5
PLT (×10^9^/L)	197.3 ± 60.7	218.4 ± 79.4	235.4 ± 89.4	201.5 ± 60.8	210.6 ± 77.7	254.0 ± 82.6
Scr (μmol/L)	66.6 ± 17.9	61.8 ± 18.2	71.8 ± 12.7	65.8 ± 27.1	64.1 ± 18.1	70.4 ± 12.4
BUN (mmol/L)	5.3 ± 1.5	7.0 ± 0.6	6.8 ± 0.6	5.2 ± 1.3	7.0 ± 0.6	6.9 ± 0.6
ALT (U/L)	18.3 ± 9.6	28.1 ± 9.4	30.0 ± 10.9	21.7 ± 13.2*	28.9 ± 10.0	29.5 ± 9.8
AST (U/L)	20.9 ± 5.5	27.8 ± 10.1	25.6 ± 9.1	23.5 ± 9.0*	28.0 ± 10.4	24.6 ± 8.2

Data are presented as mean ± standard deviation. *p < 0.05 vs. MG group at the same time point, determined by Student’s t-test. MG, monotherapy group, colchicine to dual‐therapy; CG, combination group, sustained dual‐therapy.

#### 3.2.3 Assessment of treatment safety

The tolerance profile of the dual-drug regimen was generally favorable. A marked and transient increase in the rate of drug-induced diarrhea was noted in the CG during the initial treatment stage (M1 through M3), with the incidence climbing to 35.9%, 38.1% and 39.4% at the three time points respectively; by contrast, the incidence of this adverse event remained below 1.6% throughout the entire follow-up period in the MG, with statistically significant differences observed at all three time points (all p < 0.05). Notably, no cases of drug-associated cytopenia were reported in either group across all follow-up visits. For other documented adverse effects including drug-related rash, the incidence patterns varied dynamically between the two groups over time, with a significantly lower incidence observed in the CG at M1 compared with the MG (0.0% vs. 4.0%, p < 0.05). No cases of drug allergy were recorded in either group during the study. Overall, these adverse events occurred infrequently, and no clear or sustained trend indicative of increased risk attributable to the combination therapy was observed. A comprehensive record of adverse events across all follow-up visits is summarized in **Table 5**.

**Table 5:** Comparison of side effects between the CG and MG.

Group	MG (N = 124)						CG (N = 231)					
Items	M1 no. (%)	M2 no. (%)	M3 no. (%)	M6 no. (%)	M9 no. (%)	M12 no. (%)	M1 no. (%)	M2 no. (%)	M3 no. (%)	M6 no. (%)	M9 no. (%)	M12 no. (%)
Drug-related cytopenia	0 (0)	0 (0.0)	0 (0.0)	0 (0.0)	0 (0.0)	0 (0.0)	0 (0)	0 (0)*	0 (0.0)	0 (0.0)	0 (0.0)	0 (0.0)
Drug-induced diarrhea	0 (0.0)	0 (0.0)	2 (1.6)	2 (1.6)	2 (1.6)	0 (0.0)	83 (35.9)*	88 (38.1)*	91 (39.4)*	3 (1.3)	0 (0.0)	1 (0.4)
Drug-related rash	5 (4.0)	0 (0.0)	0 (0.0)	4 (3.2)	6 (4.8)	1 (0.8)	0 (0.0)*	0 (0.0)	0 (0.0)	4 (1.7)	5 (2.2)	1 (0.4)
Drug allergy	0 (0.0)	0 (0.0)	0 (0.0)	0 (0.0)	0 (0.0)	0 (0.0)	0 (0.0)	0 (0.0)	0 (0.0)	0 (0.0)	0 (0.0)	0 (0.0)

Data are presented as number (percentage). *p < 0.05 vs. MG group at the same time point, determined by Chi-square test or Fisher’s exact test. CG, combination group; MG, monotherapy group.

## 4 Discussion

BD is a chronic systemic inflammatory disorder with complex pathogenesis and diverse clinical manifestations, among which mucocutaneous involvement is the most common and seriously impairs patients’ quality of life (1, 2). Currently, colchicine is recommended as the first-line therapy for BD patients with mucocutaneous involvement due to its significant anti-inflammatory effects (57).

In Chinese clinical practice, the combination of TGP and colchicine, clinically named “Two Flowers Therapy”, has been empirically applied for more than 30 years, and records of Paeonia-derived preparations for BD-like syndromes can even be traced back to the 1960s. Nevertheless, high-quality clinical evidence and systematic mechanistic research supporting this long-standing empirical regimen are extremely scarce worldwide. This study is the first to combine clinical cohort observation with network multi-omics analysis to comprehensively investigate the efficacy, safety, and molecular mechanisms of TGP combined with colchicine for mucocutaneous BD. This study findings not only objectively confirm the clinical value of this decades-long empirical regimen but also reveal its synergistic anti-inflammatory targets and pathways, filling the gap between traditional Chinese clinical experience and modern pharmacological evidence.

Network pharmacology and molecular docking technologies have become powerful tools for exploring the multi-component and multi-target therapeutic mechanisms of TCM (58). In this study, four main bioactive components of TGP (oxypaeoniflorin, albiflorin, benzoyl paeoniflorin, and paeoniflorin) were screened through the TCMSP database and literature retrieval, which is consistent with previous studies on the effective components of TGP (56). Through target prediction and intersection analysis, 31 overlapping targets between TGP, colchicine and BD were identified, indicating that the therapeutic effect of the combined regimen on BD may be achieved by regulating these common targets.

The PPI network analysis showed that the average degree of the 31 overlapping targets was 12.7, suggesting that these targets have strong interactions. The core targets identified based on the GEO database (MMP9, ICAM1, FGF2, TLR4, EGFR, NOS3) are closely related to the pathogenesis of BD. Specifically, our core targets, like ICAM1, TLR4, and MMP9, play key roles in relevant inflammatory pathways. The TLR 4 has been verified to be pertinent to active BD at a higher expression level and has been shown to recognize and interact with HSP and LPS, which are regarded as antigens in BD (59). Colchicine is able to downregulate expression of TLR4, which has been confirmed both in Western blot (60) and the molecular docking. In addition, research shows that active compounds in TGP can effectively suppress the TLR4/NF-κB signaling axis, a major inflammatory pathway. This directly reduces the production of pro-inflammatory factors like TNF-α and IL-6 (61, 62). In BD, these factors are central to driving blood vessel inflammation. It is reported that the adhesion molecule ICAM1, which has been demonstrated to be highly expressed in BD patients (63), is associated with aberrant leukocyte transendothelial migration (64), leading to excessive immune inflammatory response in active BD. What is gratifying is that colchicine is able to significantly reduce the expression of ICAM1, and this is also visually validated in the molecular docking. At the same time, TGP can downregulate the expression of ICAM1 on endothelial cells (65), resulting in hindering leukocytes from sticking to the blood vessel wall, directly reducing the gathering and infiltration of inflammatory cells into the blood vessel lesions of BD. As is shown in the lipid and atherosclerosis signaling pathway in KEGG analysis (**Figure 3B**), MMP9 is an enzyme that breaks down tissue and can promote the spread of inflammation, playing a vital role in cell injury and apoptosis activated by NF-κB (66); an increased level of MMP9 has also been observed in BD patients (63). Meanwhile, MMP9 participates in many inflammatory responses induced by the PI3K/Akt pathway (67). The results of molecular docking showed that the main active components of TGP (albiflorin, benzoyl paeoniflorin, oxypaeoniflorin, paeoniflorin) and colchicine could form stable binding with these core targets. Specifically, albiflorin has good binding activity with MMP9, which is consistent with previous research findings. These results suggest that MMP9 may be a key target of albiflorin in the treatment of BD, and albiflorin may exert its therapeutic effect by mediating the expression of MMP9. Thrillingly, it has already been verified that albiflorin can significantly reduce the expression of MMP9 by Western blotting (67). Moreover, colchicine also reduces the expression level of MMP9, as confirmed by Western blot (60). The synergistic downregulation of MMP9 by both albiflorin (a major active component of TGP) and colchicine indicates that the combination of colchicine and TGP may achieve more efficient therapy in treating BD. Additionally, MMP9 is a matrix metalloproteinase involved in extracellular matrix degradation and inflammatory cell infiltration, and its high expression is associated with the severity of BD mucocutaneous lesions. Notably, colchicine formed hydrogen bonds with EGFR, ICAM1 and NOS3, and had hydrophobic interactions with TLR4; albiflorin could bind to EGFR, FGF2 and MMP9 through hydrogen bonds. These results collectively indicate that the combined regimen may exert a synergistic therapeutic effect by simultaneously regulating multiple core targets related to BD inflammation.

GO enrichment analysis showed that the overlapping targets were mainly involved in biological processes such as response to molecule of bacterial origin, nitric oxide biosynthetic process, nitric oxide metabolic process, and reactive nitrogen species metabolic process. These processes are closely related to the inflammatory and immune responses of BD. BD is considered to be related to infection-induced immune disorders, and the response to bacterial origin molecules may be one of the triggers of BD inflammation (68). NO is an important inflammatory mediator, and abnormal NO metabolism can lead to excessive inflammatory response and tissue damage, which is involved in the occurrence of BD ulcers and skin lesions (69). KEGG pathway enrichment analysis showed that the top 10 enriched pathways included Lipid and atherosclerosis, AGE-RAGE signaling pathway in diabetic complications, TNF signaling pathway, IL-17 signaling pathway. Among them, the Lipid and atherosclerosis pathway was the most significantly enriched. Previous studies have found that BD patients have abnormal lipid metabolism, and lipid peroxidation products can induce inflammatory responses (70). The TNF signaling pathway and IL-17 signaling pathway are classic inflammatory pathways, and their overactivation is the key to the persistent inflammatory response of BD (71, 72). These results suggest that TGP combined with colchicine may exert a therapeutic effect on BD by regulating multiple inflammatory pathways and immune responses.

Although the effectiveness of TGP in treating BD has been clinically confirmed in China over the past 30 years, relevant discussions have mainly remained within China and have not been sufficiently recognized or disseminated in international guidelines or studies. Therefore, based on the “Two Flowers therapy” that has been passed down orally in TCM, this study designed a clinical cohort to verify its efficacy. The clinical trial results of this study showed that after 12 months of observation, the combined treatment group (CG) had significantly lower recurrence rates of oral and genital ulcers than the monotherapy group (MG) at the early treatment stage (M1 and M2). Specifically, the recurrence rate of oral ulcers in CG was 0.0% at M1 and M2, which was significantly lower than 21.0% and 100.0% in MG, respectively; the recurrence rate of genital ulcers in CG at M1 was 0.9%, which was significantly lower than 6.5% in MG. This indicates that TGP combined with colchicine can more effectively control the recurrence of mucocutaneous lesions in BD patients in the early stage of treatment. After M3, both groups achieved complete remission of oral and genital ulcers, with no significant difference between the two groups, suggesting that both regimens can achieve stable therapeutic effects in the long term, but the combined regimen has a faster and more significant effect in the early stage.

In terms of inflammatory markers, the trajectory of ESR in the two groups was significantly different. Although CG had a higher initial ESR value at M1, it decreased sharply and was significantly lower than MG at M2, and then the ESR levels of the two groups stabilized and were comparable. This dynamic change of ESR is consistent with the recurrence of clinical lesions, indicating that the combined regimen can more effectively inhibit the inflammatory response in the early stage, which may be one of the reasons for its better early clinical efficacy. There was no significant difference in CRP levels between the two groups at all time points, which may be related to the different sensitivity of CRP and ESR to inflammatory responses in BD, or the small sample size of the study. In addition, other hematological and biochemical indicators (including blood routine and liver and kidney function indicators) of both groups remained within the normal range during the study, indicating that both regimens have good safety in terms of affecting important organ functions.

The safety evaluation results showed that the tolerance of the TGP combined with colchicine regimen was generally good. The main adverse reaction was drug-induced diarrhea, which was transiently increased in CG during the initial treatment stage (M1-M3), with the incidence rates of 35.9%, 38.1% and 39.4% respectively, while the incidence rate in MG was always below 1.6%. This is consistent with previous reports that TGP may cause gastrointestinal reactions such as diarrhea (66). However, it should be noted that this adverse reaction was transient and did not require special treatment in most cases, and the incidence rate decreased significantly after M3, indicating that patients can gradually adapt to it. There were no cases of drug-related cytopenia in either group during the entire follow-up period, which is different from the previous occasional reports of colchicine-induced cytopenia (67), which may be related to the low dose of colchicine used in this study (1 mg/day).

For drug-related rash, the incidence rate of CG at M1 was 0.0%, which was significantly lower than 4.0% of MG, and there was no significant difference between the two groups at other time points. No drug allergy occurred in either group, indicating that the combined regimen does not increase the risk of allergic reactions. Overall, the adverse reactions of the combined regimen are mild, transient and manageable, and there is no clear evidence that it increases the overall risk of adverse reactions compared with monotherapy.

In traditional Chinese medicine, BD is equivalent to the historical disease entity named “狐惑” (Hu-Huo). Accordingly, China has developed a set of distinctive therapeutic approaches that have been applied for many years, with their efficacy validated through extensive clinical practice. However, these regimens are currently limited to domestic clinical use and have not been incorporated into international or national clinical guidelines. This highlights the necessity to integrate experience-based traditional Chinese medical practices into global mainstream evidence-based medicine systems (73–76).Notably, the 100% recurrence rate of oral ulcers among MG patients at month 2 is a true real-world finding from our cohort. This result demonstrates that colchicine monotherapy has limited efficacy and cannot effectively control oral ulcers in Chinese patients with BD. Long-term clinical observations have revealed obvious disparities between real-world treatment outcomes in Chinese BD patients and the recommendations put forward by international guidelines. In routine practice, colchicine monotherapy often cannot fully meet the efficacy requirements stated in clinical guidelines and shows unsatisfactory performance in managing oral ulcers. Likewise, azathioprine and the emerging agent apremilast only produce favorable responses in a small subset of Chinese patients. Faced with such unmet clinical demands, Chinese clinicians have explored alternative treatment options over the past decades. As a result, the combined regimen of TGP and colchicine has been used empirically for 30 years. Hence, this study primarily explores the efficacy and underlying mechanisms of the “Two Flower therapy” via clinical and mechanistic analyses, hoping to offer new insights for the treatment of BD.

Nevertheless, the present study has several limitations that should be acknowledged. First, this study was designed as a single-center clinical observation with a relatively limited sample size, which may have a certain impact on the statistical power of some comparative analyses. Second, the non-randomized design with allocation based on patient willingness may introduce selection bias, which should be considered when interpreting the efficacy findings. Third, both the MG and CG received TGP treatment during the trial, limiting direct comparisons with standard therapeutic regimens. Fourth, although the follow-up period lasted 12 months, longer observation may be needed to further evaluate the long-term durability of efficacy and the potential occurrence of rare adverse events. In addition, the network pharmacology and molecular docking results provided important predictive clues for the underlying mechanism, yet further experimental verification at the cellular and animal levels is still required to confirm these regulatory effects. Future multi-center, randomized controlled designs with larger populations and extended follow-up will help to provide more robust evidence, and in-depth mechanistic studies will contribute to a more comprehensive understanding of the synergistic effects of this combination regimen.

## 5 Conclusion

The “Two Flowers Therapy” — TGP combined with colchicine — achieves significantly superior early control of mucocutaneous lesions in BD patients, with a favorable safety profile characterized by only transient, manageable diarrhea. Its synergistic mechanism involves simultaneous modulation of six core inflammatory targets (MMP9, ICAM1, FGF2, TLR4, EGFR, NOS3) and key pathways, including TLR4/NF-κB, TNF, and IL-17 signaling. For clinical practice, this regimen serves as a valuable first-line complementary option, particularly for patients with an incomplete response or intolerance to colchicine monotherapy, facilitating earlier symptom remission and reduced relapse risk. For future research, multi-center randomized controlled trials with extended follow-up are warranted to confirm its long-term efficacy and generalizability, while cellular and animal studies are needed to validate these predicted molecular targets.

## Data Availability

The data used or analyzed during the current study are available from the corresponding author on reasonable request.
